# Cardiac solution for a vascular scenario!

**DOI:** 10.1186/s42155-021-00280-0

**Published:** 2022-02-01

**Authors:** Jamal Moosavi, Somaye Ahmadi, Ata Firouzi, Parham Sadeghipour, Bahram Mohebbi, Omid Shafe, Azin Alizadeasl, Sanaz Asadian, Mehran Hoseini

**Affiliations:** Rajaei Cardiovascular Medical and Research Center, Iran University of Medical Sciences, Tehran, Iran

**Keywords:** Transcatheter closure of ascending aorta pseudoaneurysm, Aortic cannulation site, Amplatzer atrial septal defect Occluder device, ASO device

## Abstract

**Background:**

Ascending aortic pseudoaneurysms (AAPs) constitute a rare, albeit potentially dangerous, condition that occurs in up to 13% of patients after cardiac or aortic surgeries. For patients with a history of cardiac surgery, repeat thoracotomy poses additive risks. The high morbidity and mortality rates associated with the surgical management of AAPs have led to the development of transcatheter approaches.

**Case report:**

We report a case of AAP percutaneous closure at the site of aortic cannulation with an ASO device in a post-CABG 65-year-old man, who refused surgery.

**Conclusion:**

The use of the Amplatzer Atrial Septal Defect Occluder (ASO) device represents an acceptable alternative to surgery in treatment of Ascending aortic pseudoaneurysms.

## Background

The incidence of ascending aortic pseudoaneurysms (AAPs) is as low as 0.5%; however, a higher incidence rate of up to 13% was reported in a surveillance imaging series of patients after cardiac or aortic surgeries (Razzouk et al., 1993). The interval between the initial operation and the recognition of thoracic aortic pseudoaneurysms varies from 3 months to 8 years (Bashir et al., 2005). Postoperative pseudoaneurysms of the thoracic aorta can be found at the sites of aortic cannulation, aortotomy, aortic anastomosis, proximal vein graft anastomosis, and the distal anastomosis of the ascending aortic graft replacement (Razzouk et al., 1993). In up to 60% of aortic surgeries, AAPs occur at the level of the suture line after surgery (Razzouk et al., 1993). Other common potential etiologies include endocarditis and trauma (Kannan et al., 2009).

Similar to all pseudoaneurysms, those of the aorta are prone to rupture, thrombosis, distal embolization, and fistula formation, hence the danger that they potentially pose (Noble & Ibrahim, 2012). Surgical repair is recommended, although it is likely associated with significant morbidity and mortality (60% mortality in a study), especially when performed in the emergency setting. Median sternotomy is particularly hazardous in that it requires technical modification to prevent exsanguination with the exposure of the pseudoaneurysm (Dhadwal et al., 2006; D’Attellis et al., 2001). It is, therefore, advisable that aggressive diagnosis be sought and sufficient heed be paid to catheter-based interventions for the initial treatment (Dhadwal et al., 2006).

## Main text

### Case report

In January 2021, a 65-year-old man came to our institution because of an AAP after refusing medical advice to undergo surgery in another center.

The patient was hypertensive and had a history of past smoking and coronary artery bypass graft surgery (CABG) 18 years earlier with a left internal mammary artery graft on the left anterior descending artery, a radial graft on the obtuse marginal-2 artery, and saphenous vein grafts (SVGs) on the obtuse marginal-1 and diagonal arteries. He had suffered transient sudden severe chest pains for 1 min 3 months previously, followed by progressive weakness, chest pains, and dyspnea.

Upon admission to the hospital, the patient had a temperature of 37.2 °C, a blood pressure of 128/72 mmHg, a heart rate of 73 beats per minute, a respiratory rate of 14 breaths per minute, and an oxygen saturation level of 95% in room air. He was alert and in good general condition.

Cardiac auscultation revealed a regular heart rhythm without any murmurs, and chest auscultation was clear. An abdominal examination showed nothing unusual. The peripheral pulses were present (2+). Laboratory results demonstrated a hemoglobin level of 13.9 g/dL, a hematocrit level of 39.3%, and a platelet count of 153,000/mm3. Additionally, urinalysis and liver function test results were normal.

**Fig. 1:**
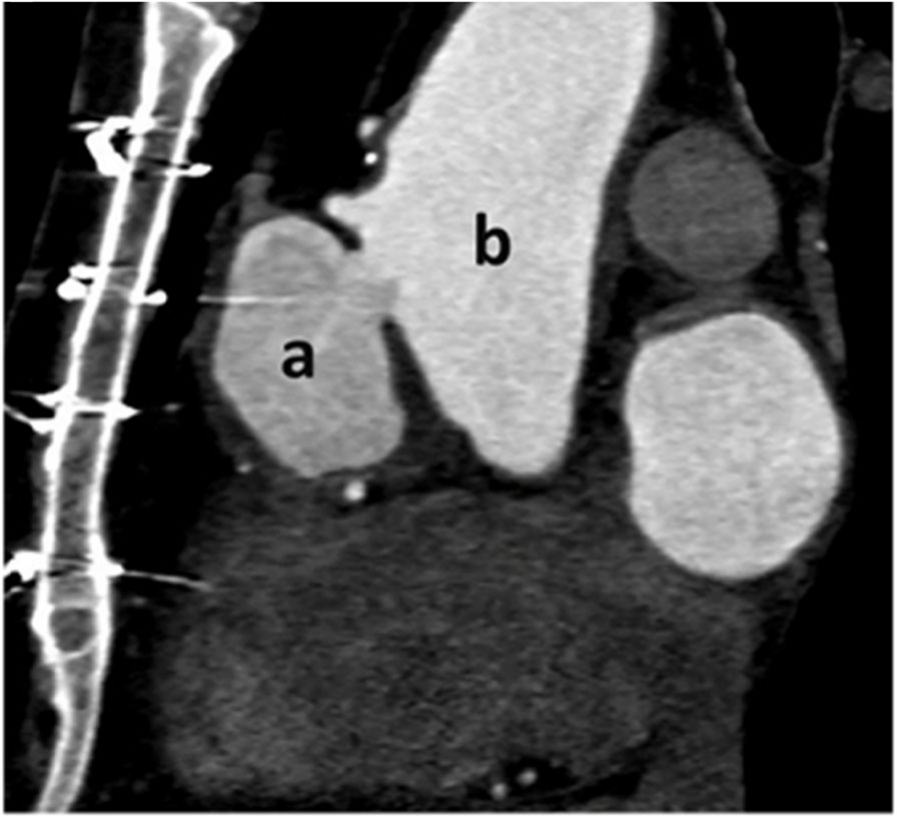
Electrocardiogram-gated CT angiography in oblique coronal plane depicts a contrast-filled outpouching from the proximal ascending aorta just above the sinotubular junction in favor of Pseudoaneurysm (**A**) Pseudoaneurysm, (**B**) ascending aorta

Aortic computed tomography angiography with contrast demonstrated a contrast-filled and outpouching wall, 44 × 33 mm in thickness, with mural irregularity. The wall protruded from the anterior aspect of the proximal portion of the ascending aorta, in favor of a pseudoaneurysm (neck diameter = 17 mm) at the substernal location just before the proximal anastomosis site of the first SVG (occluded) (Fig. 1).

**Fig. 2:**
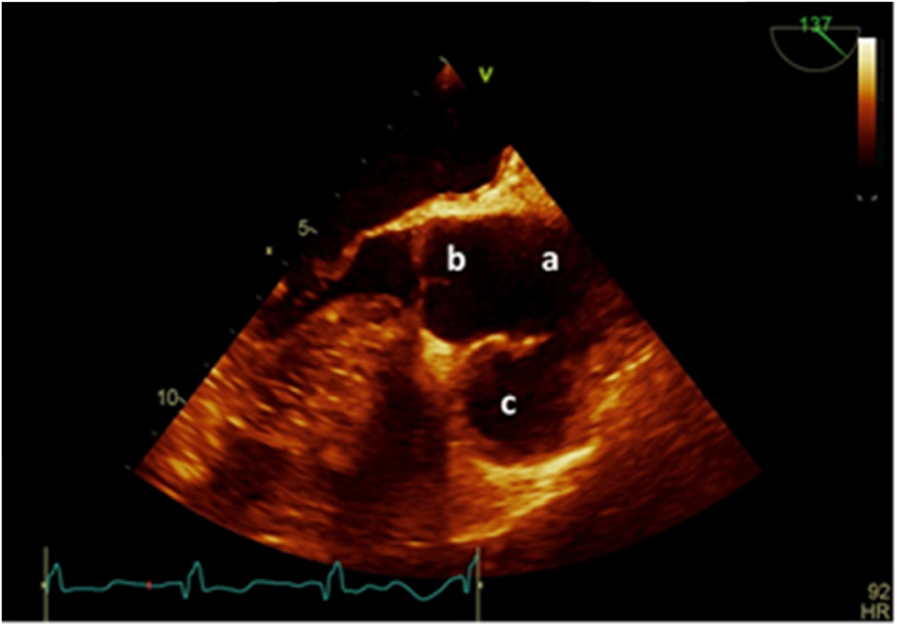
Trons esophageal echocardiography revealed Large pseudoaneurysm at right side of Aorta root (**A**) ascending, (**B**) sinus of Valsalva, (**C**) Pseudoaneurysm

A transthoracic echocardiogram revealed a left ventricular ejection fraction of 40%, a top-normal-sized (3.4 cm) ascending aorta, and a large (3.2 cm) echo-free space adjacent to the anterior wall of the ascending aorta just after the sinus of Valsalva connected with a 9 mm neck to the ascending aorta, suggestive of a large aortic pseudoaneurysm (Fig. 2).

### Technique

**Fig. 3:**
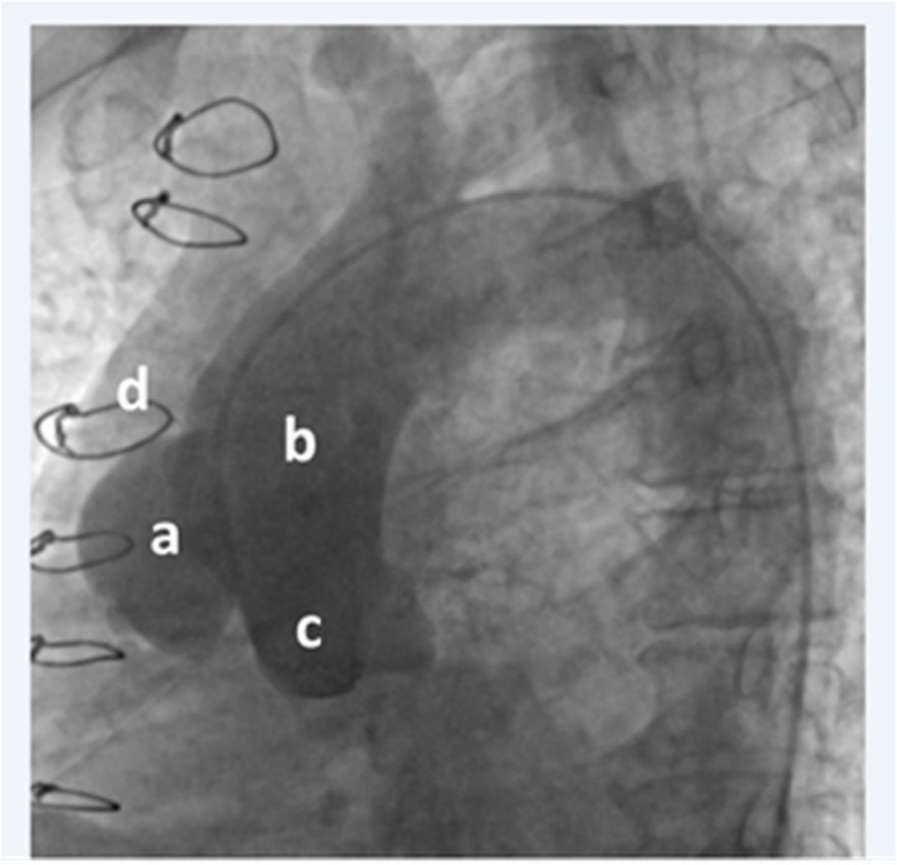
Flouroscopy during aortic root injection showed a huge pseudoaneurysm, probably at previous surgical clamp site. **A** Pseudoaneurysm, (**B**) ascending aorta, (**C**) sinus of Valsalva, (**D**) suture of sternotomy

In the catheterization laboratory, arterial access was obtained via the right and left femoral arteries. Then, two 6 F sheaths were placed in the access sites, and one 6 F pigtail catheter was advanced on a 0.035″ J-tipped guidewire via the left femoral access to the ascending aorta under fluoroscopic guidance. The contrast material was injected via the pigtail catheter to define the location of the pseudoaneurysm (Fig. 3). Thereafter, one 6 F Judkins Right (JR) Catheter was advanced on a 0.035″ J-tipped guidewire via the right femoral access to the ascending aorta and positioned within the pseudoaneurysm. The tip of the pigtail catheter was left in the ascending aorta, and it was connected to the pressure system. Subsequently, the 0.035″ J-tipped guidewire was withdrawn from the JR Catheter and exchanged with a 150 cm super-stiff guidewire, which was fixed in the pseudoaneurysm. Afterward, the JR Catheter was removed.

**Fig. 4:**
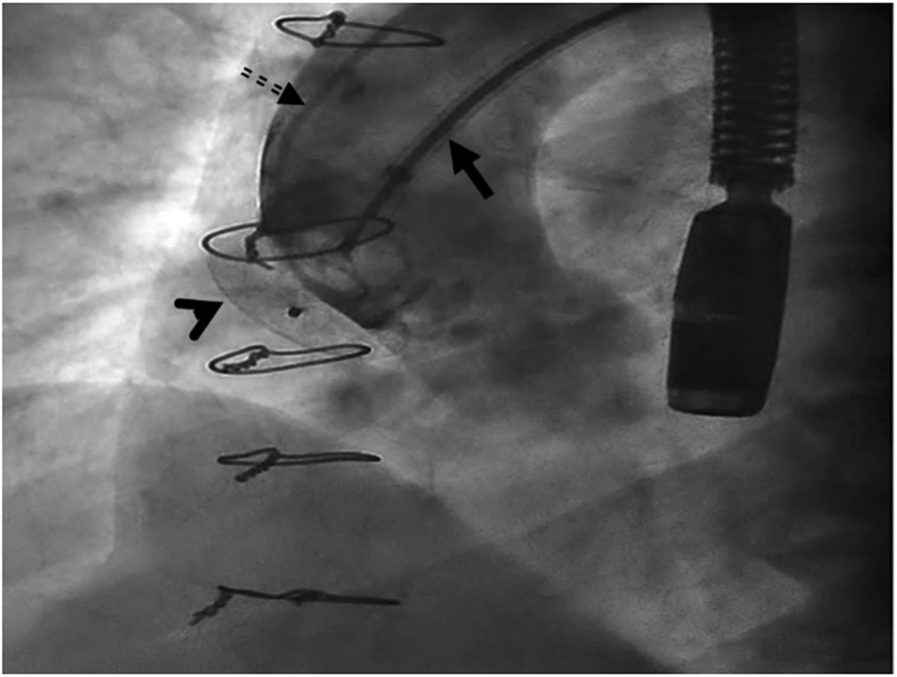
Flouroscopy during aortic root injection Before complete release of ASD occluder, revealed good position of device and total occlusion of pseudoaneurysm inlet. Arrowhead: ASD occluder. Arrow: device cable. Dash arrow: Pigtail catheter for aortic root injection

**Fig. 5:**
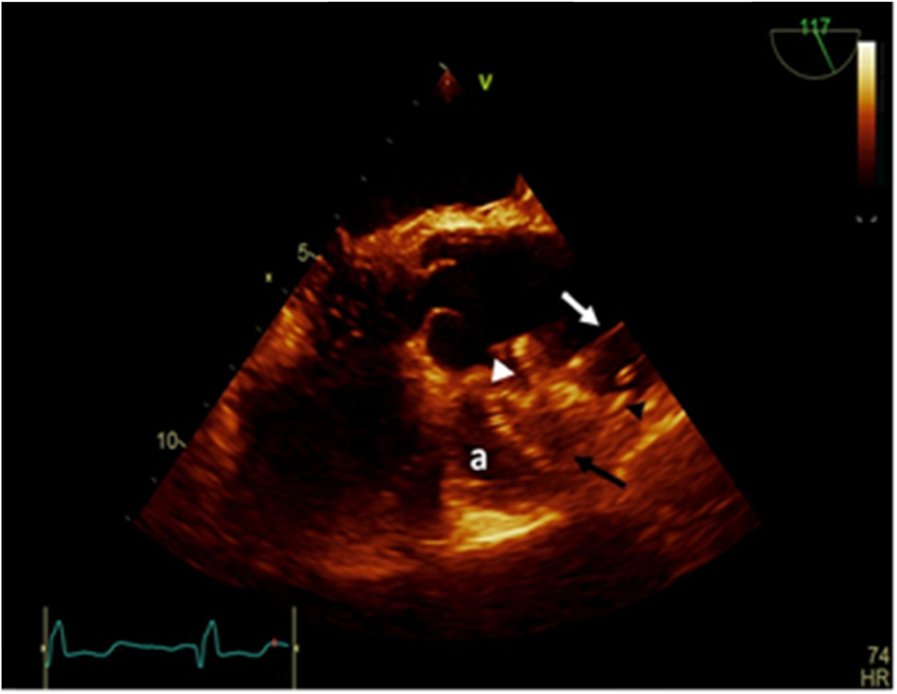
Inra-opperative Trans-esophageal echocardiography. Completed transcatheter closure of pseudoaneurysm neck with Amplatzer ASD occluder device 18 before it. **A** pseudoaneurysm. White arrow head: pseudoaneurysm neck Black arrow head: RA disk. Black arrow: LA disk white arrow: device cable

**Fig. 6:**
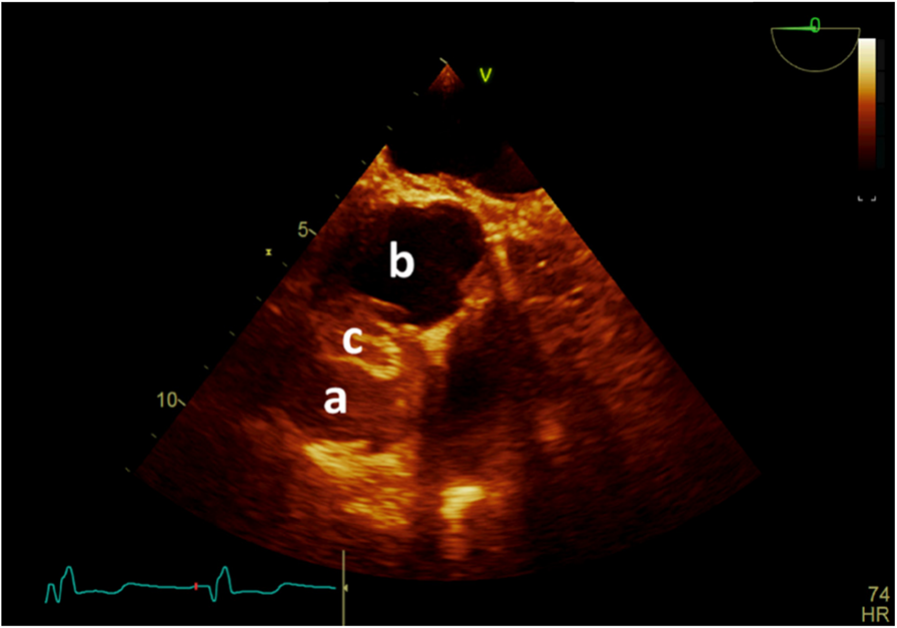
Inra-opperative Trans-esophageal echocardiography. Completed and released device with good position and no residue. **A** pseudoaneurysm, (**B**) ascending aorta, (**C**) ASD device occluder

**Fig. 7:**
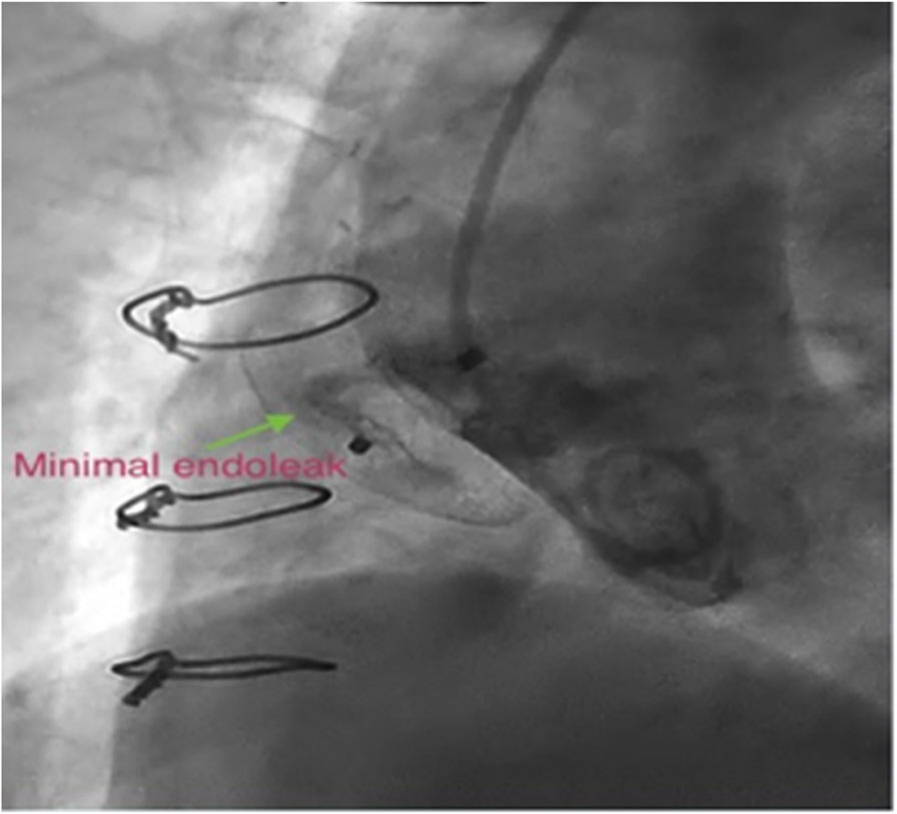
In final angiogram, a minimal extravasation remained which disappeared in following non-invasive imagings

The next stage saw the preparation of an 18 mm ASO device, as well as its delivery cable and loader, in the same manner as atrial septal defect closure procedures. The right femoral sheath was then removed and exchanged with a long 12 F sheath. The sheath was advanced on the super-stiff guidewire, with its tip within the pseudoaneurysm cavity, before its dilator and the super-stiff guidewire were removed. Next, the loading device was attached to the delivery sheath. Under fluoroscopic and transesophageal echocardiography (TEE) guidance, the device was advanced carefully to minimize contact with the wall of the cavity. When the device reached the tip of the delivery sheath within the pseudoaneurysm, the left atrial disk was deployed under fluoroscopic and TEE guidance by retracting the sheath over the delivery cable. Good apposition was achieved against the rim of the aortic tissue at the edge of the defect. Subsequently, with tension on the delivery cable, the sheath was retracted further to deploy the right atrial disk, parallel with the aortic wall. Before the release of the device, appropriate position and flow limitation were confirmed through an interrogation of all rims with TEE and contrast injection via the pigtail catheter. A fluoroscopy study demonstrated trivial flow from the aorta to the pseudoaneurysm (Fig. 4), and a Doppler interrogation suggested the complete closure of the pseudoaneurysm (Fig. 5). With gentle manipulation, the device was released and the delivery system was removed. Afterward, no complications, including pericardial effusion and compressive effects on the contiguous structures, were illustrated by TEE (Figs. 6,7).

Follow-up: A follow-up echocardiographic examination showed that the device was in the appropriate position with the complete closure of the opening between the pseudoaneurysm and the ascending aorta.

**Fig. 8:**
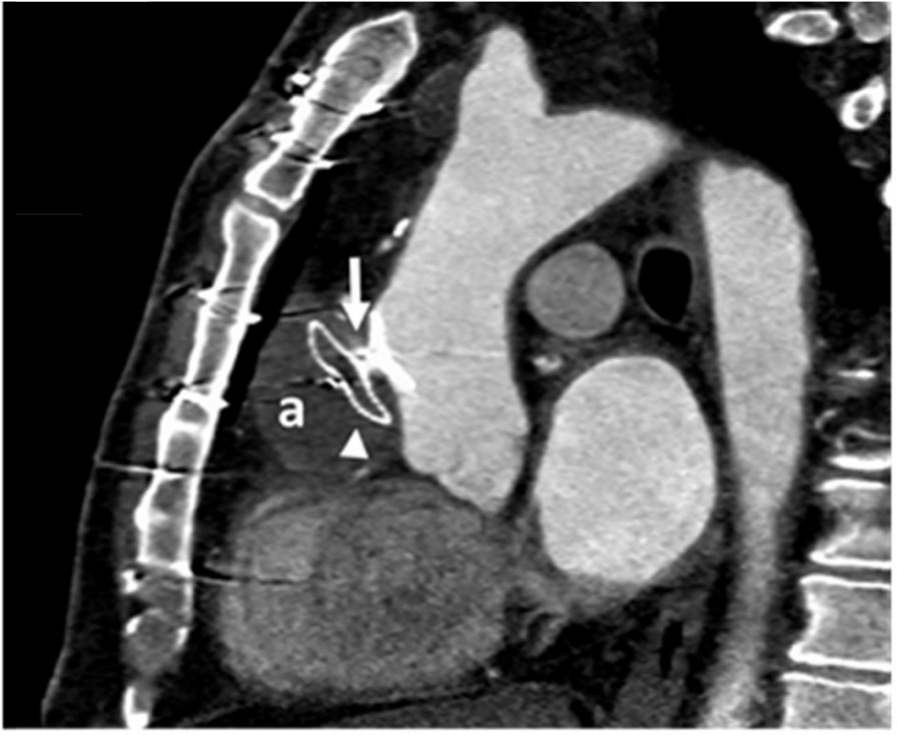
Electrocardiogram-gated CT angiography in the oblique sagittal plane in the same patient, one months after successful device closure demonstrates complete thrombosis of the pseudoaneurysm sac. **A** completely thrombosed pseudoaneurysm sac. Arrow head: device arrow: completely occluded pseudoaneurysm neck

Electrocardiogram-gated CT angiography in the oblique sagittal plane in the same patient one month after successful device closure demonstrated complete thrombosis of the pseudoaneurysm sac (Fig. 8).

## Discussion

The first report of a percutaneous method for AAP closure was published in 2005 by Bashir et al. (Bashir et al., 2005), who placed a 32 mm ASO device to treat a patient with a large AAP, considered too high-risk for surgery. Follow-up magnetic resonance imaging (MRI) and echocardiographic studies showed adequate device positioning and defect closure. TTE and MRI after six weeks showed that the device was in proper position with complete closure of the pseudoaneurysm (Bashir et al., 2005).

Henry C et al. (Quevedo et al., 2014) in 2014 performed endovascular closure with a 12 mm ASO device (St Jude Medical) for a 79-year-old man with a 9 × 10 × 7 cm AAP in the anterior mediastinum, regarded as too high-risk for surgery. CT angiography at three months’ follow-up showed a thrombosed AAP with minimal residual shunting (Quevedo et al., 2014).

A literature search of articles published in the English, Spanish, and Portuguese languages from January 1980 to May 2014 yielded 355 patients with AAPs, of whom 73.8% underwent surgical repair. In contrast, 21.2% of the patients were treated by using endovascular techniques only, either with stent-grafts (9.8%), coil embolization (1.1%), and thrombin injection (0.5%) or with occluder devices (9.8%). Further, ASO devices or vascular plugs were implanted in 36 patients, of whom 75% had successful immediate periprocedural results. The results vis-à-vis follow-ups were inconsistent, with some articles reporting the failure of the device to maintain its position over time. The authors hypothesized that the device was not properly oversized at deployment or an appropriate firm rim of tissue to anchor the device was absent. Moreover, in the cases with device malposition, the concerning features included the persistence of residual shunting into the AAP for more than 6 weeks after deployment or the continued postprocedural expansion of the AAP exceeding 2 weeks (Quevedo et al., 2014).

Kanani et al. (Kanani et al., 2007) placed an ASO device for the treatment of a 68-year-old post-CABG woman with an AAP at the site of a previous SVG anastomosis, deemed too high-risk for surgery. As was expected, a post-percutaneous closure TEE study showed minimal flow through the center of the device. Two days later, TEE demonstrated successful closure of the pseudoaneurysm and pseudoaneurysm thrombosis (Kanani et al., 2007).

Also they presented a 78-year-old post-sternectomy woman with an anterior AAP, posterior to he sternal graft. She underwent successful closure of the pseudoaneurysm using an 18-mm ASO device, with angiographic and TEE guidance. Follow-up CT angiography after one month confirmed continued occlusion and a decrease in the size of the pseudoaneurysm (Kanani et al., 2007).

Bhava R.J.Kannan (Kannan et al., 2009) reported two cases: the first, a 60-year-old man with a large pseudoaneurysm arising from the ascending aorta. Two years ago, he underwent surgical insertion of a Dacron interposition tube graft because of acute dissection of the ascending aorta. The defect was successfully occluded by an Amplatzer muscular ventricular septal defect (VSD) occluder. A CT scan, 72 h later, confirmed the correct position of the device and the complete occlusion of the pseudoaneurysm neck, and he was asymptomatic 6 months later.

The second case was a 63-year-old woman, with a history of multiple surgeries. She had had a large AAP rising from the site of a previous aortic cannulation, presented with a slowly enlarging pulsatile mass on the upper right parasternal region. She underwent insertion of an 18-mm Amplatzer muscular VSD occluder. Three months later, aortogram and CT scan showed no residual flow into the pseudoaneurysm, the pulsatile mass over her chest was completely disappeared, and she was asymptomatic (Kannan et al., 2009).

The high surgical risk associated with AAPs has led to the introduction of such novel approaches as endovascular grafts and the catheter delivery of thrombin. The off-label practice of using occluder devices for AAP closure has been restricted to patients with histories of multiple cardiac surgeries and is, thus, considered unsuitable for other thoracotomy procedures (D’Attellis et al., 2001). For patients with prohibitive operative risks, the use of the ASO device represents an acceptable alternative to surgery. The ASO device (AGA Medical, Golden Valley, MN) has been successfully used to treat atrial septal defects (ie, a somewhat parallel anatomical configuration of a gap in a thin-walled structure with 2 large chambers on each side).

## Conclusions

AAPs constitute an infrequent, albeit potentially grave, condition. For patients with a history of cardiac surgery, repeat thoracotomy imposes additive risks. The high morbidity and mortality rates associated with the surgical management of AAPs have led to the development of transcatheter approaches.

We herein presented a case of an AAP at the site of an SVG anastomosis, treated with an ASO device as an alternate strategy to surgery. The size of the defect was determined by computed tomography angiography on the ascending aorta, followed by TEE and fluoroscopy in the catheterization laboratory. TEE was also drawn upon for device placement intraprocedurally and residual flow detection across the defect postprocedurally. A follow-up echocardiographic examination and 6-month follow-up CT-angiography confirmed the appropriate position of the device and the complete closure of the opening between the pseudoaneurysm and the ascending aorta.

## Data Availability

The authors confirm that the data supporting the findings of this study are available within the article.

## Abbreviations

AAP: Ascending aortic pseudoaneurysm
ASO: Amplatzer Atrial Septal Defect Occluder
VSD: ventricular septal defect
